# The Attractiveness of Masked Faces Is Influenced by Race and Mask Attitudes

**DOI:** 10.3389/fpsyg.2022.864936

**Published:** 2022-05-17

**Authors:** Veronica Dudarev, Miki Kamatani, Yuki Miyazaki, James T. Enns, Jun I. Kawahara

**Affiliations:** ^1^Department of Psychology, University of British Columbia, Vancouver, BC, Canada; ^2^Faculty of Humanities and Human Sciences, Hokkaido University, Sapporo, Japan; ^3^Department of Psychology, Fukuyama University, Hiroshima, Japan

**Keywords:** sanitary mask, protective mask, COVID-19, facial attractiveness, microvalence, affective appreciation, affective devaluation

## Abstract

This study tests the influence of wearing a protective face mask on the perceived attractiveness of the wearer. Participants who identified as White, and who varied in their ideological stance toward mask wearing, rated the attractiveness of facial photographs. The photos varied in baseline attractiveness (low, medium, and high), race (White and Asian), and whether or not the face was wearing a protective mask. Attitudes regarding protective masks were measured after the rating task using a survey to identify participants as either pro- or anti-mask. The results showed that masked individuals of the same race were generally rated as more attractive than unmasked individuals, but that masked individuals of another race were rated as less attractive than unmasked individuals. Moreover, pro-mask participants rated masked individuals as generally more attractive than unmasked individuals, whereas anti-maskers rated masked individuals as less attractive. A control experiment, replicating the procedure but replacing the protective masks with a partially occluding notebook, showed that these effects were mask-specific. These results demonstrate that perceived attractiveness is affected by characteristics of the viewer (attitudes toward protective masks), their relationship to the target (same or different race), and by circumstances external to both (pandemic).

## Introduction

The theory of microvalences proposes that every-day objects are assigned emotional values that influence our perception and action (Lebrecht et al., 2011, 2012; Todd and Manaligod, 2018). The idea is that even the most mundane objects accrue a valence through our experience with them that is more than just random variance around a neutral mean (Lebrecht et al., 2012). This is supported by growing evidence that, just as highly-valenced objects such as guns and roses can attract our attention and guide our choices, the microvalences of common objects can do the same (Lebrecht et al., 2012; Lebrecht and Tarr, 2012; Roos et al., 2013). One untested prediction of the theory, as far as we are aware, is that the individual microvalences of objects encountered in the same context can affect one another (Lebrecht et al., 2012). If this is occurring implicitly, then person perception (the evaluation of one object) may be influenced by the microvalence of an object associated with that person (the evaluation of a second object), even if the presence of that object does not express the person’s *choice*. That is, seeing someone with a cake or a credit card in hand may increase the emotional attitude toward the person, while seeing one with a knife or a bag of garbage may decrease liking of the person. In the present study, we test this prediction by exploring how the microvalence of a newly familiar object can influence the emotional appraisal of a person using it.

One great challenge to testing this theory is that the microvalence of an object is shaped by one’s personal history of interacting with it (Lebrecht et al., 2012), which means that most microvalences are personally idiosyncratic and therefore hard to control in research. The recent worldwide COVID-19 pandemic, however, introduced a new object into the lives of many people around the world—the protective mask (Institute for Health Metrics and Evaluation, 2021)—providing an opportunity to study the microvalence associated with it in people from different populations and circumstances.

Although cloth and medical masks are effective in controlling the spread of COVID-19 (Gandhi et al., 2020; Lyu and Wehby, 2020; Howard et al., 2021; Rader et al., 2021), many people oppose this practice because of personal beliefs or ideological affiliations (Aratani, 2020; Beer, 2020; Karalis, 2020; Knotek et al., 2020; Miller and Brueck, 2020). The efficiency of the masks in reducing the spread of the virus and the resistance of some people to wearing them make investigation into the emotional appraisal of masks not only theoretically interesting, but practically relevant. In a recent study, we explored the emotional appraisal of protective masks and showed that the frequency of their use during the pandemic predicted their positive emotional appraisal in the context of a study of consumer products (Dudarev et al., 2021). This *usage-liking* relationship held up even after controlling for individual differences in beliefs about the dangers of COVID-19 and for environmental exposure to COVID-19 (Dudarev et al., 2021). In the present study, we go a step further to study the effects of a protective mask on the perception of the attractiveness of its wearer.

This question has been asked before the COVID-19 pandemic. Miyazaki and Kawahara (2016) took advantage of the fact that many Japanese citizens took to wearing masks in public following outbreaks of Spanish flu. Since then, face masks are often used in Japan to prevent the spread of colds and flu. Miyazaki and Kawahara (2016) compared the attractiveness ratings given by Japanese viewers of Japanese faces covered with a protective mask, with the same faces when they were not wearing a mask. They found that masked faces were generally rated as less attractive than unmasked faces. This cost of wearing a protective mask interacted with the baseline attractiveness, such that there was a greater cost to the ratings when the faces were otherwise rated as high in attractiveness.

Miyazaki and Kawahara (2016) identified two factors at work in the attractiveness costs of masked faces. The first factor was occlusion: A mask covering the lower part of the face hides advantageous features for attractive faces (e.g., smooth skin and symmetry), at the same time, that it hides disadvantageous features for less attractive faces (e.g., skin blemishes, scars, or asymmetry). This has the effect of truncating the range of attractiveness ratings for masked compared to unmasked faces. However, this single factor did not fully explain the results. The second factor was an appreciation that, in keeping with the social norms in Japan at the time, wearing a mask was associated with potential vulnerability of the wearer to pollen allergies, a sore throat, or more serious illness. This “unhealthiness priming” effect resulted in overall decrease in attractiveness ratings for masked faces that was independent of their baseline attractiveness. This interpretation was supported by a control experiment, where masks were replaced by health-neutral cards and notebooks, and the results showed only a truncation of the range of ratings. And as further support, Miyazaki and Kawahara’s (2016) showed that when participants were asked specifically to rate the health of mask wearers (rather than their attractiveness), the results were consistent with the effects they had attributed to “unhealthiness priming.”

The same research group replicated the procedures of the 2016 study in 2020, during the height of the COVID-19 pandemic in Japan, where virtually the entire population had taken to regularly wearing mask (Kamatani et al., 2021). They hypothesized that this normalization of mask wearing would reduce the “unhealthiness priming” effect, since masks no longer held a signal regarding any individual’s personal health status and they had become a symbol representing conformity to societal norms (Nakayachi et al., 2020). Indeed, the only effect remaining in the attractiveness ratings for masked faces in 2020 was the truncated range of ratings attributable to visible occlusion of the face.

Here, we follow the paradigm and logic developed by Miyazaki and Kawahara (2016) and Kamatani et al. (2021) to study how a protective mask affects the attractiveness of the mask wearer during the height of the COVID-19 pandemic in North America. Our study design allowed us to test two hypotheses. First, we tested whether the attractiveness of a wearer of this object depends on the perceived similarity of the wearer with the viewer. We manipulated this similarity by presenting participants with facial photographs of people of either the same race as the viewer (White) or a different race (Asian). If personal similarity to the viewer influences rated attractiveness, then protective masks should increase the rated attractiveness of own-race (White) faces relative to the rated attractiveness of other-race (Asian) faces. This own-race bias can stem from any number of factors, including the improved perceptual fluency when assessing own-race faces (Alter and Oppenheimer, 2006), greater empathy for faces that are seen as more similar to the viewer (Chiao and Mathur, 2010), and the tendency to process own-race faces with a greater level of detail than other-races faces (Levin, 2000). Here, we simply used own- versus same-races face as a convenient way to manipulate the perceived closeness of rated faces to the participant.

Our second hypothesis was that the effects of the mask on the rated attractiveness of the wearer will depend on the viewer’s attitude toward the mask itself. We measured attitudes toward masks and COVID-19 protective measures in a survey administered after the face rating task, in order to index the microvalence associated with masking for each participant. We hypothesized that more positive attitudes toward masks would be associated with more positive attractiveness ratings for people wearing masks. Conversely, anti-mask sentiment would be associated with lower perceived attractiveness of mask wearers.

The study was planned in April 2021 and the data were collected between 25 April and 5 May 2021. It is important to note that at this time, mask mandates had been implemented almost everywhere in the US for at least several months. Just after we collected our data, on 13 May, the Centers for Disease Control lifted mask mandate for fully vaccinated people, yet soon afterward this decision was reversed. Thus, seeing a person with a facial mask, in April–May 2021, could hardly be interpreted as expression of uniquely pro-social behavior. Rather, we interpreted it as minimally complying with the new rules and norms.

## Experiment 1

**Figure 1:**
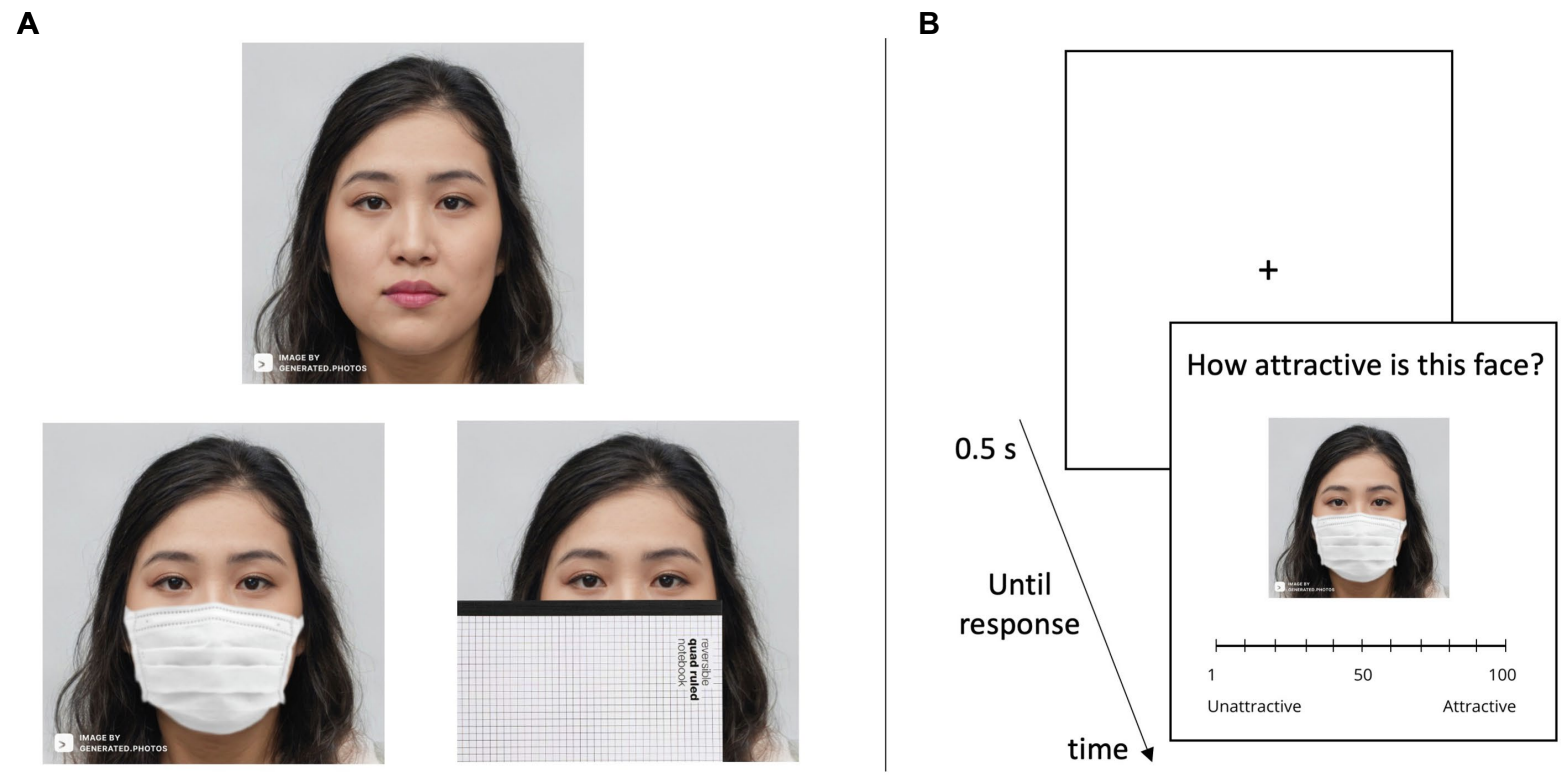
**(A)** Examples of an unmasked Asian face shown together with the same face when masked (Experiment 1) or when partially occluded by a notebook (Experiment 2). **(B)** The trial event sequence in the study. The image depicted in the figure was computer generated for the purposes of illustration (https://generated.photos/) and was not one of the faces used in the study.

We randomly assigned participants to one of two versions of a face attractiveness experiment: faces of the same race as the participants (White) or faces of another race (Asian). As shown in Figure 1, half of the faces in each condition, presented randomly, were wearing a white protective face mask and the other half were wearing no mask. We hypothesized that attractiveness ratings for masked faces would be relatively more positive for faces of the same race as the participant than for faces of a different race. Such an own-race advantage for masked faces could come about either because participants are better able to infer missing details from familiar faces (Carragher and Hancock, 2020; Freud et al., 2020; Molnar-Szakacs et al., 2021) or because masks on own-race faces are seen as more similar to the participant and thus deserving of empathy (Dudarev et al., 2021).

### Method

#### Participants

Based on the previous studies (Miyazaki and Kawahara, 2016; Kamatani et al., 2021), we aimed to collect data from at least 32 participants in each condition. We estimated the statistical power of our experimental design to detect the previously documented interaction between attractiveness and masking (Miyazaki and Kawahara, 2016; Kamatani et al., 2021), based on a sample size of 32 and an effect size of $\eta_{p}^{2}\;$ =0.23, was approaching 1. Our estimate of the power to detect an interaction between race and masking, based on two independent groups of 32 participants for each race of faces, was 0.82 for a large effect ( $\eta_{p}^{2}\;$ = 0.13) and 0.42 for a smaller effect ( $\eta_{p}^{2}\;$ = 0.06; Lakens and Caldwell, 2021).

Anticipating potential data loss due to participants’ failure to complete all the trials, inattention to the task, or failure to follow instructions, we set our recruitment sample size to be 36 participants per condition. We also automated the random assignment of participants to conditions (race of faces) through MTurk, which meant that there was no guarantee the final sample size would be exactly equal.

Participants self-identifying as White and aged 18 or older were recruited on Amazon’s MTurk platform.^1^ Participants’ race was determined *via* an eligibility survey that included demographic questions about their ethnicity, family income, age, and gender. Another requirement for participation was that the experiment was conducted on a desktop or laptop computer.

#### Materials

The photographic images used in the study consisted of female faces. The Asian faces were of Japanese individuals used in a previous study of face attractiveness (Miyazaki and Kawahara, 2016). This set of images consists of 48 photographs of young adult women with either neutral or slightly smiling expressions. The images were rated for attractiveness by a group of Japanese participants, as described in Miyazaki and Kawahara (2016), and were subdivided into three highly reliable categories of attractiveness: low, medium, and high (see Supplementary Material for details). The White female faces used in the study were taken from the Chicago Face Database (Ma and Wittenbrink, 2015).^2^ Only versions with neutral expressions were used. The images in this database already have normed attractiveness ratings, which allowed us to select 48 images, evenly divided to represent low, medium, and high levels of attractiveness. Descriptive statistics on the images selected for the present study from norming studies (Ma and Wittenbrink, 2015; Miyazaki and Kawahara, 2016) and the present study are available in the Supplementary Material. To obtain 96 corresponding masked faces, we digitally edited each of the photos in order to cover the lower portion of the face with a white protective mask. We pasted the same mask image into each face after adjusting it for size and smoothing artifactual edges using a Gaussian blur tool (Adobe Photoshop CS6).

#### Procedure

Figure 1 shows examples of the face images in the study and illustrates the trial event sequence. During the attractiveness rating task, participants were presented with a face on each trial that was either wearing a protective mask or not. Participants were instructed to rate the attractive of this face on a 100-point scale (1 = most unattractive and 100 = most attractive). A line depicting the scale from 1 to 100 was shown below each face and participants responded by clicking the line in the location corresponding to their rating. Participants were not given any time limit for their responses.

In several practice trials, prior to testing, participants were shown a very attractive face from the set that was not used in the study, as well as an extremely unattractive face. This was done in order to help them calibrate their responses to the full range of the scale and to reduce idiosyncratic individual differences.

Images of faces covered with a mask and faces wearing no mask were presented in random order. Each participant only saw a given individual’s face one time, either masked or not masked, and these selections were made randomly by the computer program so that each participant rated a total of 48 individuals, evenly distributed over the three attractiveness levels.

Participants were randomly assigned to view either White or Asian faces by the computer program. We chose a between-participant design for two reasons. First, we did not want participants to knowingly compare their own ratings for faces of one race to ratings of another race. Rather we wanted only their spontaneous responses to faces that varied in baseline attractiveness within a single race, without possible contamination from thoughts of how they were handling cross-race ratings. Second, we wanted the study to be a brief online study, so that considerations of tediousness or fatigue would not be a factor.

After completing the rating task, participants filled out survey questions about their demographic and sociocultural status, and their attitudes, practices, and experiences with the COVID-19 virus and protective masks (Dudarev et al., 2021). The entire testing session took no longer than 30 min, and participants were paid 2$. The study was approved by the Behavioral Research and Ethics Board at the University of British Columbia (approval number H21-00305). The data were collected between 25 April and 5 May 2021.

### Results

#### The Effects of Race on Perceived Attractiveness of Masked Faces

Seventy-nine participants completed all 48 trials of the face attractiveness ratings, with 30.4% self-identified as female, and a mean reported age of 39.9 (ranging = 22–72 years). Forty-two participants were randomly assigned to rate White faces (31% female); 37 were assigned to rate Asian faces (29.7% female). Two participants (one from each group) responded with the same rating on all trials, but exclusion of their data did not change the results.

**Figure 2:**
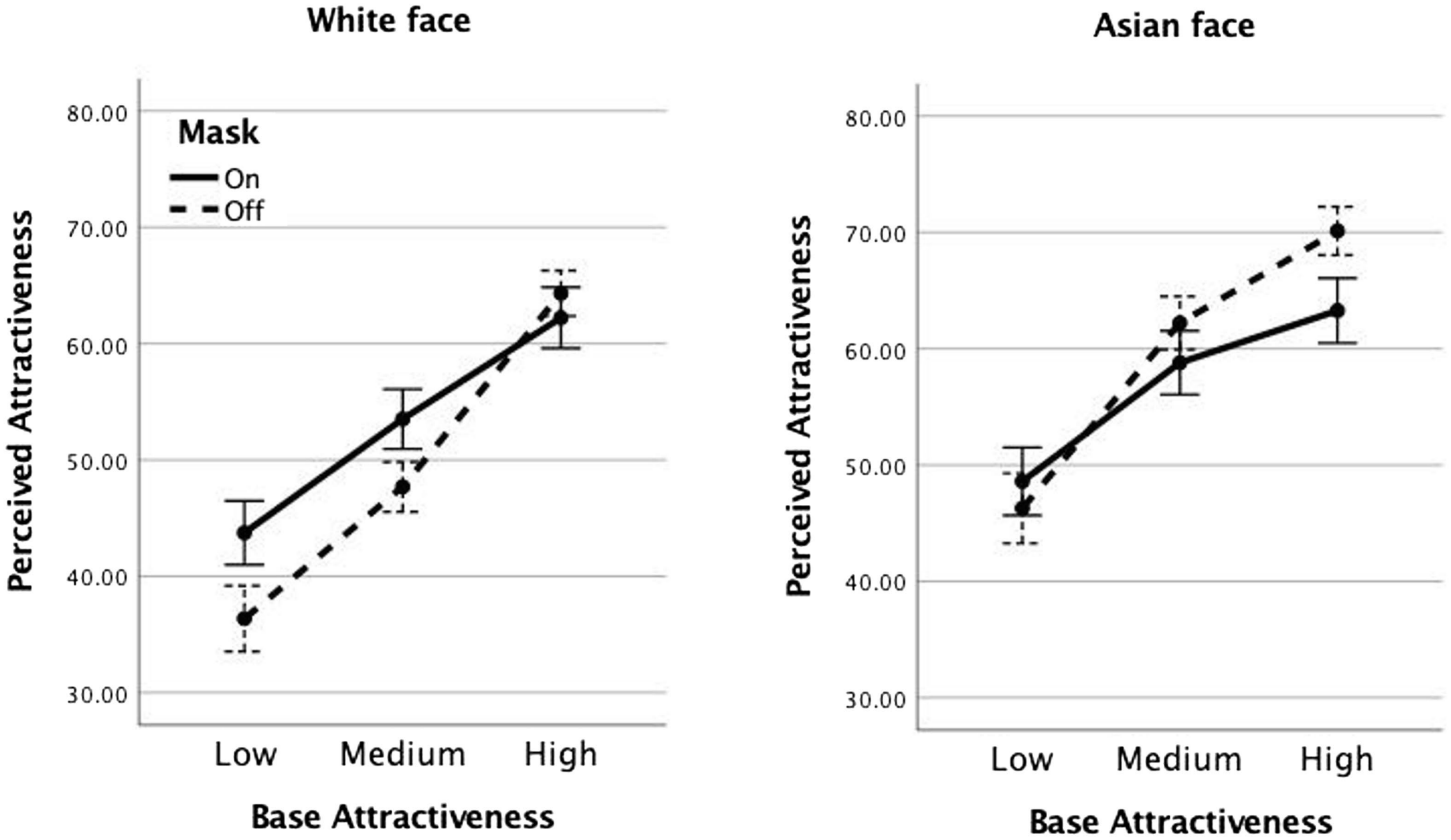
Mean attractiveness ratings for White and Asian faces of low, medium, and high base attractiveness, when covered with a protective mask (solid line) or not (dashed line). Error bars represent +/− 1 standard error.

Figure 2 shows mean attractiveness ratings as a function of the within-participant factors of attractiveness level (low, medium, and high), and mask (off and on), and the between-participant factor of race of face (White, Asian). The three levels of baseline attractiveness were established using normed data from previous studies (Ma and Wittenbrink, 2015; Miyazaki and Kawahara, 2016). A mixed model ANOVA of these ratings showed an expectedly strong effect of attractiveness, *F*(2,154) = 102.58, *p* < 0.001, $\eta_{p}^{2}$ = 0.571, replicating previous findings for these faces (White faces: Ma and Wittenbrink, 2015; Asian faces: Miyazaki and Kawahara, 2016). The main effect of attractiveness was qualified by an interaction with mask, *F*(2,154) = 21.15, *p* < 0.001, $\eta_{p}^{2}$ = 0.215, reflecting that the addition of a protective mask reduced ratings for highly attractive faces and increased ratings for unattractive faces. The main effect of mask was not significant, *F*(1,77) = 0.18, *p* = 0.67, $\eta_{p}^{2}$ = 0.002.

Race also yielded a main effect, *F*(1,77) = 5.86, *p* = 0.018, $\eta_{p}^{2}$ = 0.071, reflecting that Asian faces were rated overall as more attractive than White faces. But this main effect was qualified by an interaction with Mask, *F*(1,77) = 6.57, *p* = 0.012, $\eta_{p}^{2}$ = 0.079, reflecting that masked Asian faces were rated lower than unmasked Asian faces, while masked White faces were rated higher than unmasked White faces. No other effects reached significance, *Fs* < 2.5, *ps* > 0.09.

To examine whether individual differences in the way participants used the attractiveness scale affected the results, we have computed a centered score by subtracting each participant’s mean rating from each rating on a single trial. The centered scores revealed the same pattern of results as the raw ratings. Note that participants’ use of the scale was calibrated during the practice trials.

**Figure 3:**
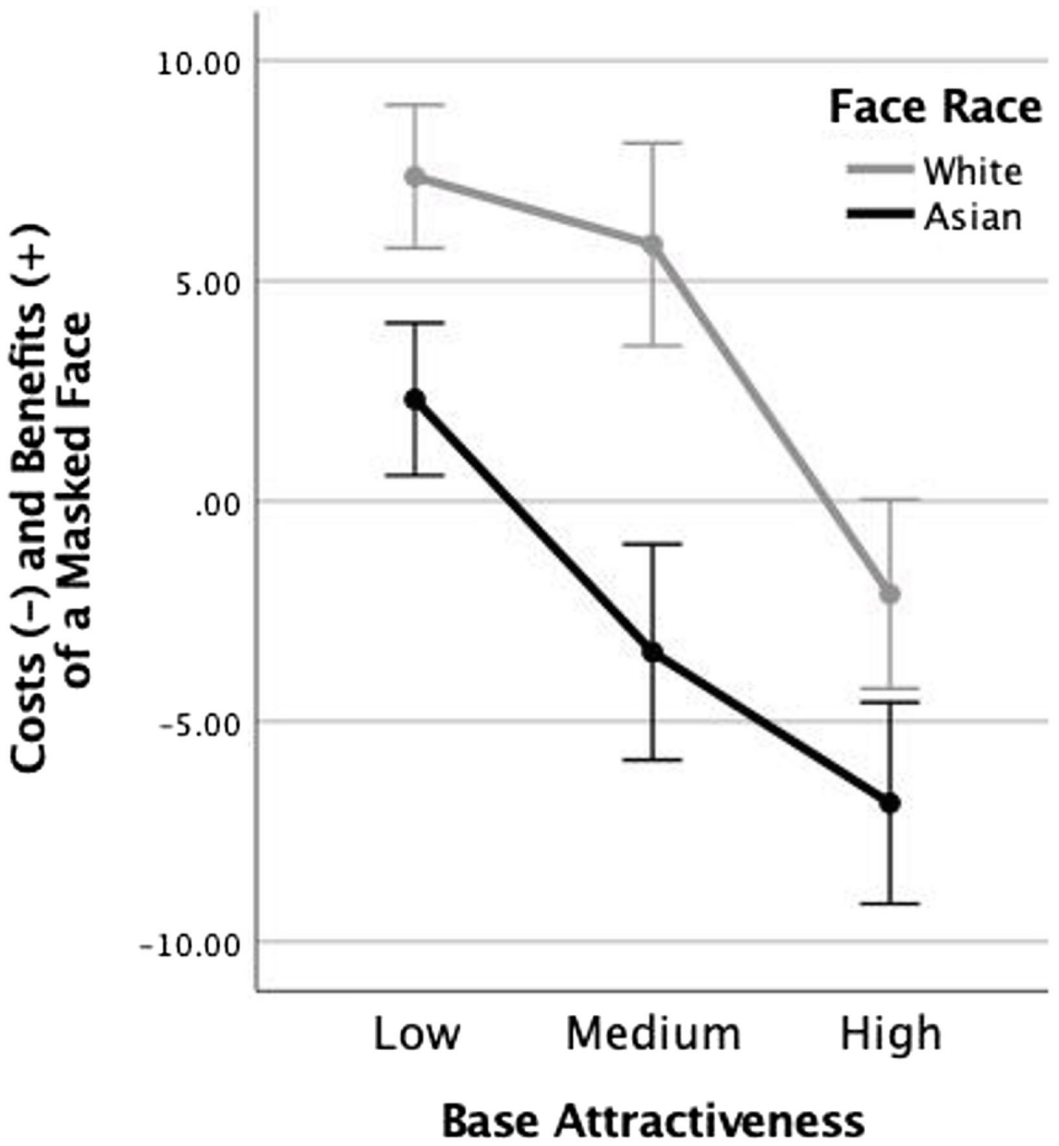
Mean difference scores show the difference between ratings for masked and unmasked faces, across the three levels of baseline attractiveness and two races. The difference scores were calculated for each participant as the difference between ratings for masked and unmasked faces. Error bars represent +/− 1 standard error.

To help visualize the nature of the race × mask interaction, we summarized the costs and benefits of wearing a protective mask by subtracting each participant’s attractiveness ratings for faces with no mask from faces with a mask. The mean difference scores are shown in Figure 3 and were submitted to a mixed model ANOVA examining the within-participant factor of attractiveness level (low, medium, and high) and the between-participant factor of race (White and Asian). We note that this analysis is redundant with the previous one, the only difference is that once the mask factor is expressed as a difference score, the interaction between attractiveness and mask becomes a main effect of attractiveness, and the interaction between race and mask becomes a main effect of race. The three-way interaction between attractiveness × race × mask is now accordingly a two-way interaction between attractiveness × race. This approach to examining repeated-measures effects as simple contrasts, based on theoretical considerations, is recommended by statisticians both for its increased clarity of presentation and for its efficient handling of any concerns regarding assumptions of sphericity (Wickens and Keppel, 2004). Here, the theoretical consideration is that we are primarily interested in what wearing a mask does to face attractiveness scores.

The analysis of the difference scores showed two main effects. The main effect of attractiveness, *F*(1,77) = 21.15, *p* < 0.001, $\eta_{p}^{2}$ = 0.215, shows that wearing a mask benefits attractiveness ratings when a face is already seen as highly attractive and reduces the ratings when it is seen as lower in attractiveness. The main effect of race, *F*(1,77) = 6.57, *p* = 0.012, $\eta_{p}^{2}$ = 0.079, shows that masked Asian faces were generally seen as lower in attractiveness than unmasked Asian faces, while for the White faces, the opposite was true. The absence of any interaction between these two factors, *F*(2,154) = 1.52, *p* > 0.2, $\eta_{p}^{2}$ = 0.019, highlights that the race and attractiveness effects on the ratings are independent of one another.

### Discussion

The results of Experiment 1 first of all replicate an important finding reported by Kamatani et al. (2021) concerning the consequences of wearing a mask on face attractiveness ratings. Faces that are reliably rated low in attractiveness when viewed in full are rated as more attractive when masked; highly attractive faces are rated as less attractive when partially covered with a mask. Along with Kamatani et al. (2021), we interpret the truncated range of attractiveness ratings for masked faces to the occlusion of visible evidence that places these faces at each end of the attractiveness scale. Facial blemishes and flaws that contribute to low ratings are less visible, as are the features supporting high ratings of attractiveness at the other end of the scale.

The novel finding from Experiment 1 is that attractiveness rates of faces are influenced differently by the addition of a protective mask, depending on the similarity of the race of the face to the viewer’s own race. Adding a mask to the White faces improved their rated attractiveness, while adding the same mask to Asian faces decreased their attractiveness ratings. It is worth considering two possible explanations for this effect. One possibility is that people are better at distinguishing identities and emotional expressions of faces of their own race than faces of a different race (Elfenbein and Ambady, 2002; Brielmann et al., 2014), possibly because scanning patterns are different for faces of different races (Brielmann et al., 2014). On this account, the occlusion of the face that accompanies the mask may have a differential influence on own-race versus other-race faces because viewers are better able to infer missing details from familiar faces (Carragher and Hancock, 2020; Freud et al., 2020; Molnar-Szakacs et al., 2021). Own-race faces that are more familiar will benefit in their rated attractiveness from the addition of a mask, whereas other-race faces that are less familiar will receive reduced ratings.

A second possibility is that protective masks carry an additional emotional value for participants—a microvalence that is positive for some viewers and negative for others (Dudarev et al., 2021). A microvalence attached to a protective mask may differentially influence ratings of faces that are seen to be more relevant to the participant (own race) than faces that are less personally relevant (other race). In Experiment 2, we devised a test to distinguish between the possibilities by occluding the lower portion of the faces with a notebook rather than a protective mask. Our assumption was that a notebook would be seen as more emotionally neutral than a protective mask by viewers during the pandemic. If notebooks produced similar results for those obtained in Experiment 1 for protective masks, it would support the interpretation that occlusion of the lower portion of the face has a differential influence on own- versus other-race faces. However, if the results using notebook occlusion eliminated the other-race effect seen in Experiment 1, then it would support the microvalence account. This account proposes that the emotional value associated with protective masks affects own- and other-race faces differently because of an interaction between the emotional appraisal of the mask and the personal relevance of the face wearing the mask.

## Experiment 2

### Method

Experiment 2 followed the procedures of Experiment 1 in their entirety, with a single change. Half of the faces now had their lower portions covered with a notebook (as shown in Figure 1) rather than a protective mask.

#### Participants

Seventy-five participants completed the experiment (29.3% female, mean age = 40.5, ranging from 22 to 72 years), with 37 of them randomly assigned to rate White faces (32.4% female) and 38 to rate Asian faces (26.3% female). None of the participants in Experiment 2 had participated in Experiment 1. Two participants (one from each group) responded with the same rating on all trials, but exclusion of their data does not change the results.

### Results

These data were analyzed in the same way as in Experiment 1. The mixed model ANOVA on the ratings showed only a main effect of attractiveness, *F*(2,146) = 84.87, *p* < 0.001, $\eta_{p}^{2}$ = 0.538, and an interaction between notebook occlusion and attractiveness, *F*(2, 146) = 29.04, *p* < 0.001, $\eta_{p}^{2}$ = 0.285. Notebook occlusion had neither a main effect nor was involved in any other interactions, *Fs* < 1, *ps* > 0.7. These results are similar to the effects produced by mask occlusion in both magnitude and pattern (see Supplementary Figure S1). However, a crucial difference in these data involving notebook occlusion was that there was not a significant interaction between race and occlusion, *F*(1,73) = 1.88, *p* = 0.17, $\eta_{p}^{2}$ = 0.025.

**Figure 4:**
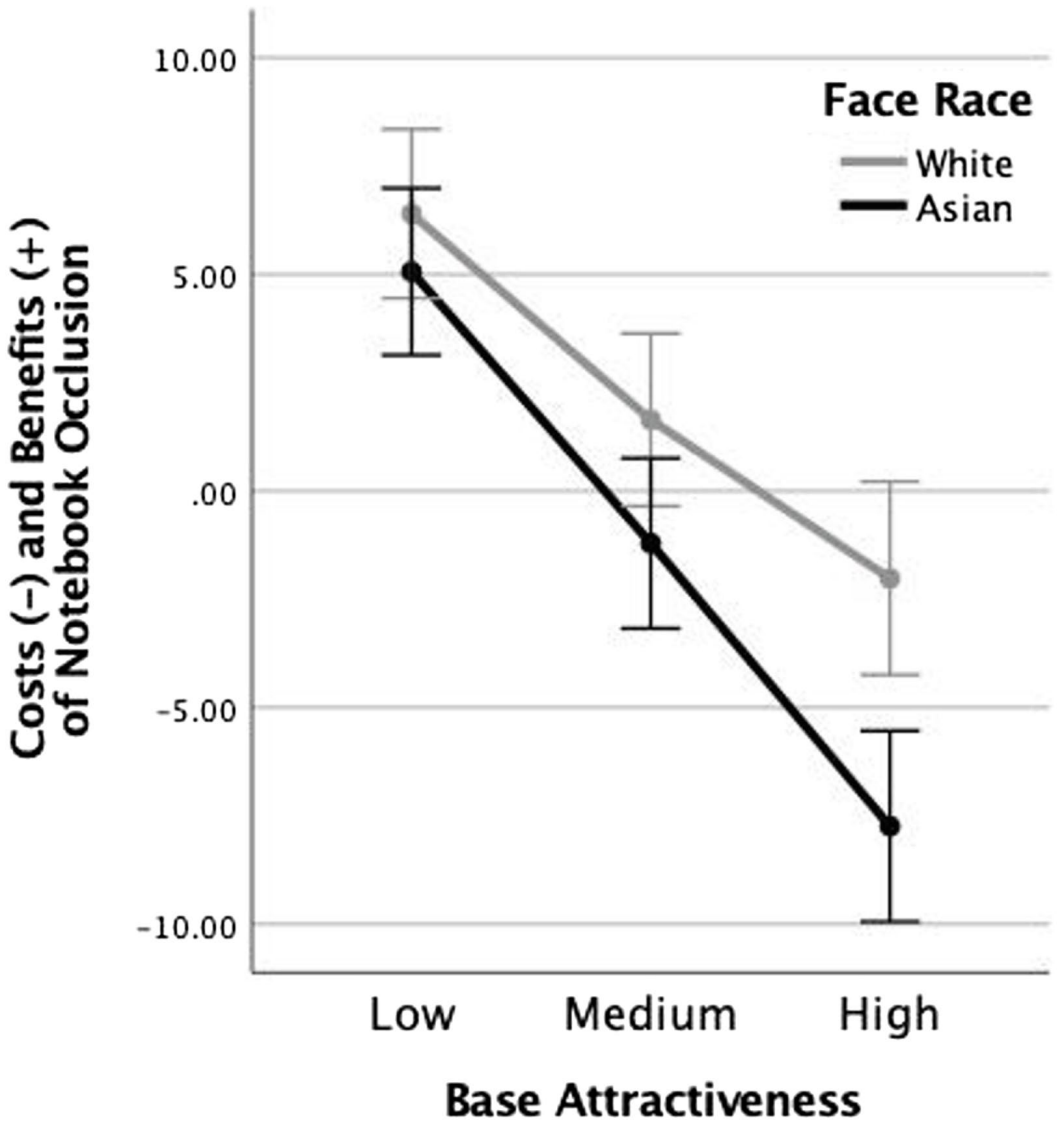
Notebook occlusion cost and benefit score, computed as the difference between attractiveness ratings for faces occluded with a notebook and those not occluded by anything, for White and Asian faces. Error bars represent +/− 1 standard error.

Figure 4 shows the differences scores summarizing the benefits and costs of being rated for attractiveness when the lower portion of the face is occluded by a notebook. Like the masked faces in Experiment 1, notebook occlusion differentially affected faces that varied in baseline attractiveness, *F*(2, 146) = 29.04, *p* < 0.001, $\eta_{p}^{2}$ = 0.285, specifically increasing ratings of low-attractive faces and reducing ratings of high-attractive faces. However, there was no effect of race in the notebook occlusion differences scores, *F*(1,73) = 1.88, *p* = 0.174, $\eta_{p}^{2}$ = 0.025. The interaction was not significant either, *F*(2, 146) = 1.28, *p* = 0.28, $\eta_{p}^{2}$ = 0.017.

**Figure 5:**
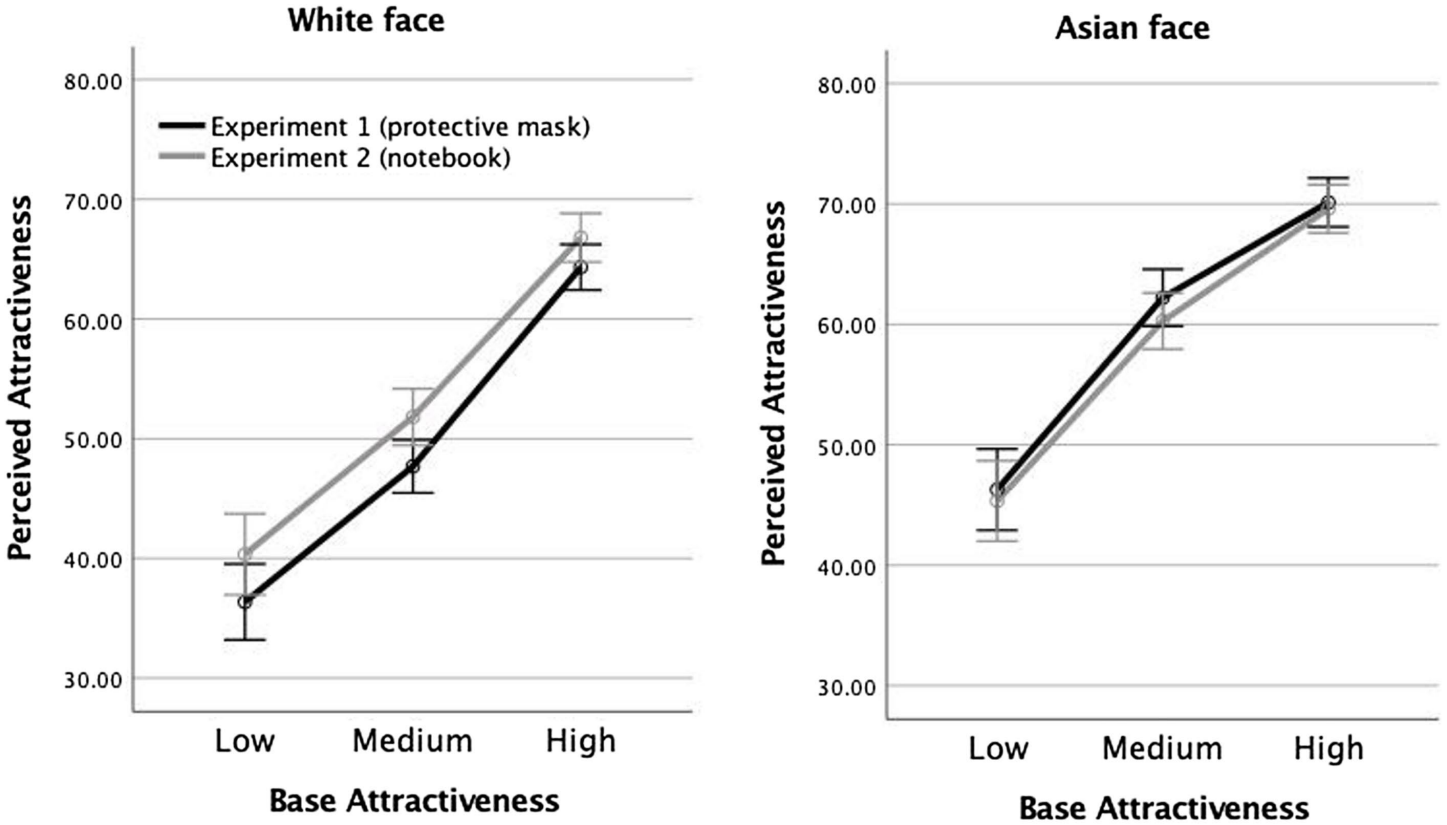
Perceived attractiveness for faces without any occlusion in Experiment 1 (black line) and Experiment 2 (gray line). Error bars represent +/− 1 standard error.

Could it be that the different pattern of results in Experiments 1 and 2 reflected group differences in the way attractiveness was being evaluated? To test for this possibility, we focused on the parts of Experiments 1 and 2 that were most similar, namely, the ratings of full faces viewed without any occlusion. These data are shown in Figure 5 and they confirm that baseline attractiveness ratings were comparable in the two experiments. A 3 (base attractiveness) × 2 (race) × 2 (Experiment: 1 vs. 2) ANOVA revealed that the factor of Experiment had no main effect, *F*(1,150) = 0.306, *p* = 0.58, $\eta_{p}^{2}$ = 0.002, and was not involved in any interactions, *Fs* < 1.2, *ps* > 0.2. Thus, the pattern of results we observed when faces were partially covered with a protective mask versus a notebook cannot be attributed to baseline differences in the way these different groups of participants rated the attractiveness of faces in general.

Race, on the other hand, had a main effect, *F*(1,150) = 12.97, *p* < 0.001, $\eta_{p}^{2}$ = 0.08, which was qualified by an interaction with base attractiveness, *F*(2,300) = 4.004, *p* = 0.019, $\eta_{p}^{2}$ = 0.026. The interaction arose from participants assigning a smaller range of ratings to other-race than same-race faces.

### Discussion

The results of Experiment 2 fully supported the microvalence account of the results of Experiment 1. Partially occluding faces with a notebook did not result in the own-race effect that occurred when faces were partially occluded with protective masks. We interpret this as supporting the view that both protective masks and other human faces are implicitly associated with emotional evaluations that influence our perceptions and actions. We will return to this interpretation below; in our analysis of the results from the attitude survey, we conducted with all participants.

But first, it is worth considering the consequences of partial face occlusion on attractiveness ratings. This refers to those results that were held in common between Experiments 1 and 2. As proposed by Miyazaki and Kawahara (2016) and Kamatani et al. (2021), partially occluding a face in any way has the consequence of truncating the range of attractiveness ratings, resulting effectively in a regression to the mean. This means it improves the ratings of low-attractive faces and reduces the ratings of high-attractive faces. And when the occlusion was attributable to an emotionally neutral object, its moderating effect was similar for own- and other-race faces.

At the same time, we acknowledge that occlusion by notebook versus mask differs not only in emotionally relevant ways, but also in a number of lower-level stimulus features. For example, the notebook in Experiment 2 occluded a slightly larger portion of the face than the mask in Experiment 1, possibly leading to greater truncation of ratings across the three levels of attractiveness. Notebook occlusion was also less symmetrical, a factor which might decrease overall ratings of attractiveness. These issues have been addressed to some extent in previous research. Miyazaki and Kawahara (2016) compared attractiveness ratings made to faces occluded with a notebook (Experiment 3a, 3b) with faces occluded with a paper card that was more similar in its size and its symmetry to a protective face mask (Experiment 4). The data were clear in showing that notebooks and cards produced very similar effects (Experiments 3 and 4) and that both types of occlusion resulted in a general improvement in attractiveness ratings, compared to faces occluded by protective masks. Thus, previous data collected in a pre-pandemic setting suggested that the size and symmetry of the occlusion did not play a measurable role in attractiveness ratings. And when it came to a comparison between these accidental types of occlusion and the wearing of a protective mask, facial occlusion by masks generally improved attractiveness ratings.

We were also able to address these concerns in our own data, collected during the same period of time and from the same participant population as the protective masking data. The full statistical analysis of the notebook occlusion data along with the graph of these data that correspond to Figure 2 for the mask data is reported in the Supplementary Material. The main take-away from this full analysis is that occlusion by notebook did not result in any discernible enhancement nor decrease in attractiveness ratings when compared to occlusion by mask. Thus, both previously reported data and the data presented here suggest that the stimulus differences that remain between notebooks and protective masks do not affect face attractiveness. Neither do they account for the different patterns in the attractiveness ratings for faces of different races.

## The Influence of Mask Attitudes on Perceived Attractiveness of Faces Wearing Protective Masks

Microvalence account posits that emotional appraisal of objects is determined, among other things, by participants’ personality and individual history. According to this interpretation, participants’ ideological attitudes toward COVID-19 protective measures should be a mediating factor in determining the rated attractiveness of people wearing masks. It is now well known that political and medical advice to wear protective masks during the pandemic sharply divided people in the United States. Our intent was therefore to elicit participants’ attitudes regarding society’s response to the pandemic and regarding various proposed measures of protection, which we did in the post-test survey to avoid contaminating our dependent variable (attractiveness ratings).

In a previous study using the same survey items (Dudarev et al., 2021), the results showed that attitudes expressed by participants about COVID-19 measures were better predictors of their emotional appraisal of consumer products associated with the pandemic (e.g., protective masks, hand hygiene products, and protective gloves) than of similar items that were not related to COVID-19 (e.g., ski goggles, shampoo, and winter gloves). In the present study, we therefore hypothesized that participants who identified as pro-mask would show a greater increase in attractiveness ratings for mask-wearers than participants who identified as anti-mask. Our secondary question was whether these mask-related biases would also be expressed more strongly for faces seen as more personally relevant to the participants (own-race) than faces seen as less personally relevant (other-race). In other words, we tested whether the wearer’s race and the viewer’s attitudes toward masks have interactive influence on the wearer’s perceived attractiveness or whether their effects are additive.

### Attitude Survey Data

All participants in Experiment 1 and 2 were presented with the attitude and demographic survey after first the completing the rating task (136 of the participants completed it). The complete list of 48 questions is presented in the Supplementary Material. The questions included participants’ age, gender, income, profession, cultural background, and religion (Whitman et al., 2018), as well as questions about participants’ political leanings and interest in politics. In addition, participants were asked several questions focusing on their COVID-19 experiences (“Have you had COVID?”), vulnerability and exposure to the virus (“Do you consider yourself to be especially vulnerable to COVID?”; “How many people do you meet offline on a typical week?”—Wright et al., 2020), and their use of protective equipment, mainly protective masks (“These days, are you using a protective face mask on a regular basis?”; “How many masks do you have now?”). We aggregated this information by submitting participants’ answers to a principal component analysis (PCA), with the aim of discovering latent constructs that might summarize a participant’s emotional appraisal of protective masks and other COVID-19-related measures.

Table 1 shows the 28 questions that were included in the PCA, including questions on political views and interests as well as focused on COVID-19 (experiences, vulnerability, exposure, and protective practices). The overall dataset fits the requirement of PCA reasonably well, with Bartlett’s test of sphericity, $\chi^{2}$ (378) = 1819, *p* < 0.001, and a Kaiser-Meyer-Olkin measure of sampling adequacy equal to 0.75.

**Table 1:** Twenty-eight questions for PCA and their loading on the first component.

Question	Loading
Politically, would you describe yourself as left or right wing?	**−0.717**
I put on a mask when going into a store/supermarket/pharmacy	**0.713**
Do you think the danger of COVID-19 has been underestimated?	**−0.708**
Do you think the usefulness of protective face masks against COVID-19 infection has been underestimated?	**−0.707**
These days, are you using a protective face mask on a regular basis?	**0.671**
During a typical week, on how many days do you meet with people offline?	**−0.649**
On how many days in the past week have you met other people (offline)?	**−0.647**
I put on a mask every time I leave the house	**0.620**
When I’m outside, I have a mask with me	**0.592**
Politically, would you describe your close friends and family as left or right wing?	**−0.578**
Have you had COVID-19(coronavirus)?	**0.516**
These days, are you using hand sanitizer on a regular basis?	**0.458**
How often do you use a face mask?	**0.454**
Are you working from home?	**0.343**
How often are you checking the news on COVID-19?	−0.219
How often are you checking news unrelated to COVID-19?	0.106
Do you consider yourself to be especially vulnerable to COVID-19?	−0.258
I put on a mask even when I exercise	−0.012
Are you interested in politics?	−0.128
How often were you checking the news before the COVID-19pandemic?	0.046
Do you live in close contact with a person who is especially vulnerable to COVID-19?	−0.248
Do you enjoy debating political issues?	−0.233
Is a mask mandatory for your work?	0.429
I estimate the situation and only put the mask on when I think it is necessary	0.026
I put on a mask only when I am in a crowded place	0.220
Do you tend to be an anxious person?	0.250
How many masks do you have now?	0.077
Do you consider yourself an independent thinker?	0.058

*Bold font denotes items that loaded on the first of the two components.*

The first component accounted for 20.5% of the total variance and was readily interpretable as one’s general attitude toward COVID-19, differentiating between participants who believed that the danger of COVID-19 was overestimated and those who thought it was underestimated. Participants’ overall self-reported political orientation (right vs. left) loaded highly on this component, as did the question “Do you think the usefulness of protective face masks against COVID has been overestimated or underestimated?” It thus seems reasonable, for the purposes of the present study, to treat this latent variable as a scale ranging from strong anti-mask at one end to strong pro-mask at the other end.

We next computed each participant’s mask attitude score using the regression method. These scores were symmetrically distributed around a value near zero with positive scores representing pro-mask attitudes, and negative scores representing anti-mask attitudes. For ease of presentation, we performed a median split on this score (Median = −0.046) and used it as a factor in ANOVAs to examine the mask attitudes of the viewers alongside the factors of the race of the face (White and Asian) and the type of partial occlusion (protective mask and notebook). We note that regression analyses, in which these scores, were treated as a continuous variable and the factors of race and occlusion were coded as dummy predictors, led to the same conclusions.

### Analysis of the Attitude Survey Data

#### The Influence of Mask Attitudes on Ratings of Faces Occluded by a Mask

**Figure 6:**
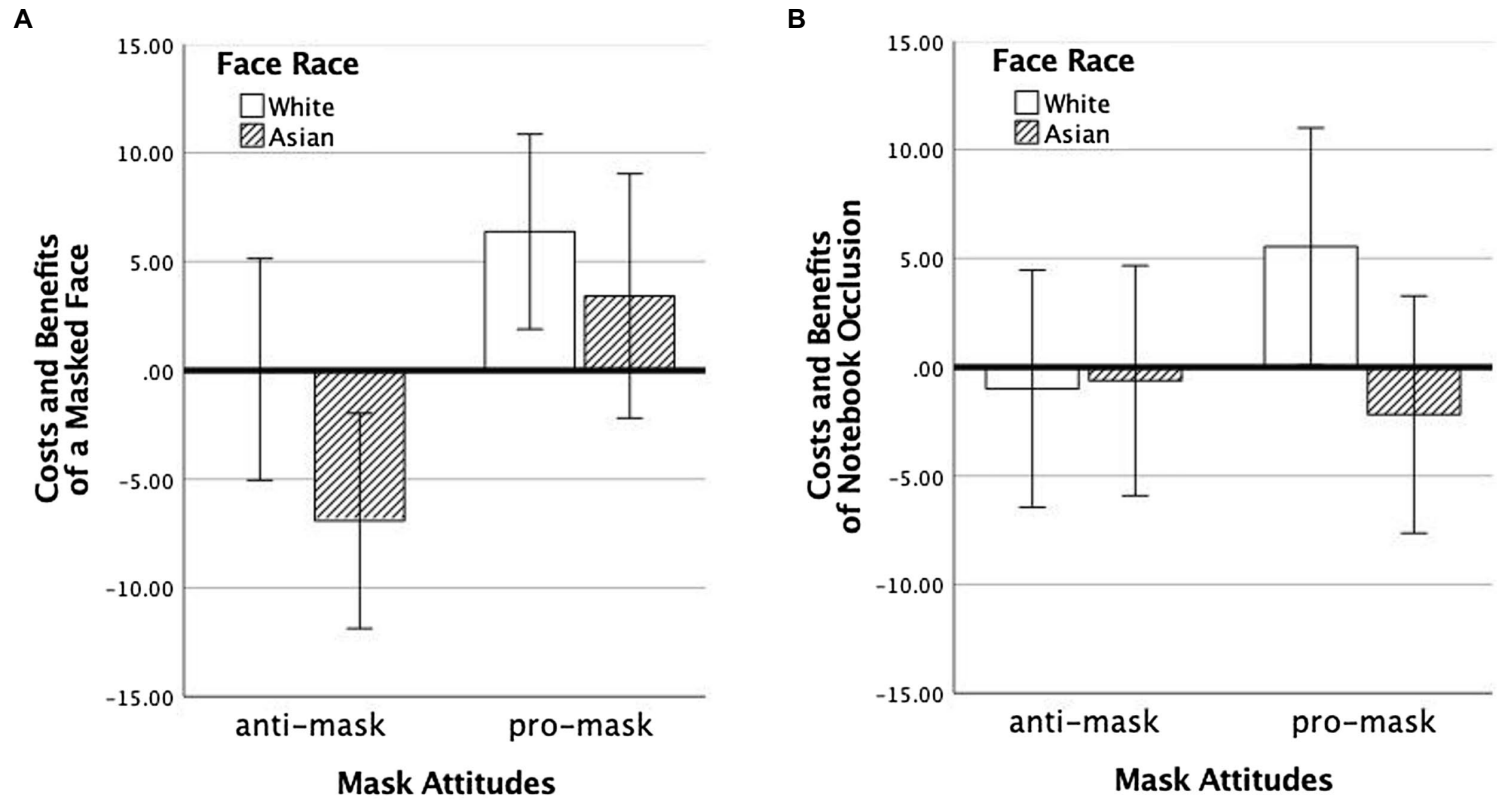
Costs and benefits of an occluded face (computed as the difference between averaged ratings for occluded and full faces, across the three levels of baseline attractiveness) as a function of the viewer’s attitudes to masks (horizontal axis) and the race of the target face (color of the bars). Panel **A** shows results for faces covered with masks; Panel **B** shows results for faces occluded by a notebook. Error bars represent +/− 1 standard error.

We first investigated the combined effects of mask attitudes and the race of the faces on participants who rated the attractiveness of faces shown with and without protective masks (Experiment 1). Because base attractiveness did not interact with race on mean rating differences scores (Figures 3, 5), we averaged each participant’s difference scores (the difference between attractiveness ratings for faces occluded with mask/notebook and fully visible faces) across attractiveness levels and examined it with an ANOVA in which mask attitude and race were the two factors. These scores are shown in Figure 6A.

The ANOVA showed a marginal main effect of race, *F*(1,67) = 3.84, *p* = 0.054, $\eta_{p}^{2}$ = 0.054, with own-race faces generally receiving higher scores than other-race faces. There was also a main effect of mask attitude, *F*(1,67) = 10.84, *p* = 0.002, $\eta_{p}^{2}$ = 0.139, with pro-mask participants giving higher scores than anti-mask participants. The interaction of these two factors was not significant, *F*(1,67) = 0.43, *p* = 0.43, $\eta_{p}^{2}$ = 0.009.

Single-sample *t*-tests were used to test for costs and benefits (i.e., differences from zero, *p* values and *CI*s are Bonferroni corrected) of the occlusion in these scores for each group of participants. Pro-mask participants showed positive biases in their scores for both own-race (White), 95% *CI* [1.83 10.92], *t*(21) = 3.83, *p* = 0.004, *Cohen’s d* = 0.817, but not other-race (Asian) faces, 95% *CI* [−0.92 7.76], *t*(13) = 2.28, *p* = 0.16, *Cohen’s d* = 0.610. Anti-mask participants, on the other hand, showed a trend toward negative bias for other-race faces, 95% *CI* [−15.60 1.77], *t*(17) = −2.22, *p* = 0.16, *Cohen’s d* = −0.524, while for own-race faces, the scores were not different from 0, 95% *CI* [−8.95 9.04], *t*(16) = 0.014, *p* > 0.36, *Cohen’s d* = 0.003.

This pattern of results suggests that when participants held positive attitudes toward masks, they also tended to give higher attractiveness ratings to all mask wearers, regardless of their race. When participants held negative attitudes toward masks, they did the reverse, tending to give lower attractiveness ratings to all people wearing them, regardless of their race. And the results show that this influence from the personal opinions of participant was additive in its effect with the race of the person wearing the mask. The practical implication of this pattern of results is that the decrease in attractiveness that derives from an anti-masking attitude, when combined with the general effect of a bias against masked Asian faces, means that half of the participants showed a negative bias toward mask wearers of the other race.

It should be noted that participants’ attitudes toward masks were closely correlated with their political affiliations. Specifically, right-leaning participants generally disliked protective masks and had doubts about their efficiency. Therefore, it could be political affiliation that drives the bias against masked Asian faces observed here. Yet previous studies suggest that attitudes toward masks and esthetic judgments of them are affected not only by political affiliation (Dudarev et al., 2021), but by frequency of wearing the masks and situational exposure to COVID-19. This is why we chose to use the aggregate of all potential variables to assess participants’ overall attitudes toward masks, instead of trying to distinguish among the possible determinants. The question of whether it is the political views alone or other aspects of mask attitudes cannot be addressed in this correlational data set, as these two issues are closely intertwined.

Another important question is whether attitudes toward protective masks are stable within an individual, and how changing attitudes over time affect the perception of masked faces. A previous study conducted by some authors in our group indicated that mask attitudes in a similar North American sample of participants did not change significantly within a three-month period during the height of the pandemic, from July to October, 2020 (Dudarev et al., 2021). However, it is reasonable to expect that changing circumstances or even merely a longer period of exposure to masked faces might be associated with change in the attitudes. Tracing the trajectory of changing attitudes toward masks on the perceived attractiveness of mask wearers is thus an important question for future studies to consider.

#### The Influence of Mask Attitudes on Ratings of Faces Occluded by a Notebook

We next examined the combined effects of mask attitudes and race of the faces for participants who rated faces that were partially occluded by notebooks (Experiment 2). These results are shown in Figure 6B. This analysis revealed no main effects and no interactions, all *p* > 0.13. The only significant single-sample t-test for the four groups of participants in Experiment 2 was for pro-mask participants, who showed a positive bias when viewing own-race faces, 95% *CI* [1.54 9.55], *t*(15) = 3.93, *p* = 0.004, *Cohen’s d* = 0.981 (all other *p* > 0.4).

## General Discussion

The present study tested the consequences of wearing a protective mask on facial attractiveness during the worldwide COVID-19 pandemic. Our scientific interest in this question derives from the theory of microvalences, which proposes that our actions and perceptions are subtly influenced by the automatic emotional appraisal we give to objects (Lebrecht et al., 2012; Lebrecht and Tarr, 2012; Roos et al., 2013). These authors point out that even common, every-day objects are not emotionally neutral. Rather, the automatic emotional appraisal we attribute to objects, derived from our past experiences with them, influences the actions we make toward them. For example, we more readily approach positive objects and tend to avoid negative objects (Chen and Bargh, 1999; Kramer et al., 2020).

The specific focus of the present study was on potential interactions between the microvalenced appraisals people give to protective masks and the appraisals of people who wear them. To examine this question, our study looked at two factors that might mediate the effect of protective masks on facial attractiveness. One factor was the perceived similarity of the faces being rated to the participant (same- vs. other-race). We hypothesized that people who appeared to be more similar to the participant (same race) might gain an attractiveness benefit when donning a mask, compared to people who appeared to be less similar (other-race). The second factor was the participants’ stance toward protective masks themselves. Wearing a protective mask became quite a contentious personal issue for many people in North America as the pandemic unfolded, with some people strongly identifying as pro-mask and others as anti-mask. It is not within the scope of this paper to reiterate the many factors leading to this societal divide. For the purpose of the present study—testing microvalence theory in a real-world context—it was simply convenient to be able to use these pre-existing individual differences in valence for masks as a way to examine object-person interactions in ratings of facial attractiveness.

Our practical interest in testing these hypotheses concerns the societal consequences of mask wearing and person perception. Given the rise in anti-minority sentiment, and specifically anti-Asian acts of violence and aggression that accompanied the pandemic in North America (Center for the Study of Hate and Extremism, 2021; Choi, 2021), it is important to better understand some of the factors that influence person perception in a health crisis such as the COVID-19 pandemic. Previous studies have documented that Muslim head and face coverings reduce the rated attractiveness of the wearer and the participant’s empathy toward them (Saroglou et al., 2009; Mahmud and Swami, 2010; Unkelbach et al., 2010). Yet it is unclear whether these effects reflect stereotyping of an out-group (Saroglou et al., 2009; Mahmud and Swami, 2010) or the emotional valence of the head-coverings themselves. In any case, it is important to remind the reader that protective masks were worn for health reasons by people worldwide during the COVID-19 pandemic. This did not allow masks to act as stereotyping signal for specific religious or ethnic groups. Despite this, the present data show that masks have a disproportionate negative influence on the perception of minority individuals, and this negative influence is compounded by pre-existing negative attitudes regarding protective masks.

It will be an important question for future studies to try and identify the specific factors that are at play in the other-race effect we see here for mask wearing. These factors may range from having less sensitivity to the face parameters of other races (Rhodes et al., 2009), to failing to treat faces from other races with the individuality afforded to same-race faces (Roos et al., 2013), to processing other-race faces less holistically (Roisson and Michel, 2011), to biases that render other-race faces less attractive (Burke et al., 2013) or less relevant to ourselves (Scott and Monesson, 2009). Regardless of the underlying factors, the present finding makes it important for both health professionals and politicians to be aware of the consequences of taking decisions such as public mask mandates on minority individuals. At a minimum, a health-focused measure such as a mask mandate should also take into account the negative consequences of masks for minority wearers. More proactive thinking dictates that additional public measures (e.g., public messaging and empathy building media portrayals) should be taken to mitigate the negative socio-political consequences of health-related decisions.

Before turning to the present findings that speak most directly to microvalence theory, as it applies to protective masks, it is important to establish two important background effects that influence attractiveness ratings when the lower portion of a face is occluded. One of these effects is not mask-specific; the other is mask-specific. A non-mask-specific effect of lower face occlusion is that it truncates the range of attractiveness ratings that are obtained for the same faces when they are seen in full. This finding has been reported by two independent research groups (Patel et al., 2020; Kamatani et al., 2021) and it was replicated again in the present Experiment 2, where we used a notebook to occlude the lower portion of the faces. The effect manifests as a simple regression to the mean of all ratings, such that highly attractive faces are rated as less attractive with a mask, and unattractive faces are rated as more attractive. Our interpretation of this effect is that occlusion limits the available evidence concerning both extremes of attractiveness; hiding the imperfections that otherwise lead to lower ratings as well as the outstanding features that would generate the highest ratings if they were visible (Kamatani et al., 2021).

In this context, it is also of interest that the range of attractiveness ratings given to other-race full faces in this study were truncated compared to the range of ratings given to same-race full faces. This can be seen by comparing the range of attractiveness rating functions for White vs. Asian faces in Figure 5. A possible interpretation of this finding is that when faces consist of features we are less familiar with, it becomes harder to assess the extreme ends of the attractiveness scale. Similar to visible occlusion, unfamiliar facial features limit the evidence available to participants for rendering a strong judgment in favor of either end of the scale.

It could be argued that this difference stems from the fact that Asian faces in this study had either neutral or slightly smiling expressions, while all the White faces study had strictly neutral expressions. It is indeed possible that the slight smiles in the Asian faces could have contributed to the slightly higher mean ratings of attractiveness given to them versus to White faces (see Figure 2). However, it is less likely that those smiles contributed to the reduced range of ratings given to Asian versus White faces by the white participants in our study (see results of Experiment 2 and Figure 5). And it is even more difficult to sustain the argument that those slight smiles were responsible for the specific effect of masks which manifested in reducing ratings at all three levels of baseline attractiveness for Asian faces when compared to White faces (the main effect seen in Figure 3).

A mask-specific effect that has been previously documented also provides important background for the present findings. A study conducted in pre-COVID-19 times by Miyazaki and Kawahara (2016) first documented that, in addition to the truncation of attractiveness ratings, protective masks contribute to an “unhealthiness priming” effect that decreases the wearer’s attractiveness overall. That is, in addition to the occlusive effects of a mask, there was a specific health-related stigma that reduced attractiveness ratings for all faces, but especially faces in the medium and high range of the baseline attractiveness scale. This reduction did not extend to a control experiment where masks were replaced by health-neutral cards and notebooks. In 2020, the same group showed that health-related stigma disappeared, as during COVID-19 wearing a protective mask was not perceived as a sign of healthiness of the wearer (Kamatani et al., 2021).

These two previous findings—truncated ratings for occluded faces and a health stigma for protective masks—thus provide a critical backdrop for the two main present findings. First, there was a same-race benefit along with an other-race cost for mask wearers. Second, participants who identify as pro-mask gave an attractiveness benefit in their ratings to individuals of both same and other races wearing masks. This contrasted with anti-mask participants, who assigned an attractiveness cost specifically to the ratings of individuals from another race. We next consider each of these two main findings in turn.

The same-race benefit and the other-race cost of mask wearing on attractiveness ratings cannot be readily interpreted as merely another instance of the truncation of ratings for occluded and for less familiar faces. If that were the case, we would not expect to see such a large benefit in attractiveness ratings for both low and medium attractive faces that were masked (see Figure 3: White faces). Clearly, something more than regression to the mean is influencing these results for White viewers of White faces. Our initial hypothesis is that this reflects the generally positive evaluation of the mask during the pandemic “leaking” into the attractiveness ratings of the wearer. Similarly, something more than regression to the mean is influencing the cost of mask wearing for Asian faces (see Figure 3: Asian faces). Here too, the negative effects of masks go beyond simple regression to the mean. Now protective masks take on a negative stigma, perhaps more related to personal dissimilarity and a lack of empathy than to reduction in evidence for attractiveness. These two points are both supported when we consider the notebook control data in Experiment 2 (see Figure 4). Note that the regression to mean effect that accompanies partial face occlusion is more similar for same- and other-race faces, supporting the truncation in attractiveness ratings that accompanies the mere absence of evidence.

This interpretation of the other-race effect for mask wearers is only strengthened when we consider the mediation of these effects by a participant’s pre-existing ideological stance regarding mask wearing (see Figure 6A). Participants who were pro-mask offered an attractiveness benefit in their ratings to individuals of both races while those who were anti-mask participants assigned an attractiveness cost specifically to the ratings of other-race faces. And again, this pattern was much reduced when the partial occlusion of the face was attributable to a non-COVID-19-related factor: notebooks (see Figure 6B). These findings clearly point to protective masks not being emotionally neutral objects in the context of person perception. The attractiveness of a face is clearly influenced, in both positive and negative ways, by the microvalences that have become associated with the objects associated with a face.

In summary, the present study demonstrates once again that protective masks were microvalenced for North American participants during the pandemic, both positively and negatively (Dudarev et al., 2021). However, the present study goes a step further in documenting that these object-based microvalences have important consequences for attractiveness ratings of a person wearing a mask. This finding surely advances our understanding of microvalence theory, showing for the first time that the emotional appraisal of objects encountered in the same context (here masks and people) can influence one another (Lebrecht et al., 2012). However, it also holds important background information for public officials in health and government charged with making decisions for the population as a whole. As a first step, the data show that the consequences of health-related decisions (i.e., mask mandates) have differential effects on mask wearers from majority and minority communities.

It will be important in future research to study cultural differences in the response to worldwide adoption of protective masks. Readers should note that while personal attitudes toward mask wearing are polarized in North America at this time, the same is not true in some Asian countries. For example, altruistic intentions to act collectively against the pandemic have strongly motivated mask wearing in Japan (Nakayachi et al., 2020). As such, the two factors shown to influence masked-face attractiveness ratings in the present study—an individual’s similarity to the rated face and their attitudes toward masks—may not contribute to the perceived attractiveness of mask wearers in Japan. This certainly awaits further examination.

## Data Availability Statement

The datasets presented in this study can be found at: https://zenodo.org/badge/latestdoi/394767599.

## Ethics Statement

All study procedures were approved by Behavioral Research and Ethics Board at the University of British Columbia (approval number H21-00305). The participants provided their written informed consent to participate in this study.

## Author Contributions

MK and YM processed the photographs and created stimuli. VD programmed the study and collected and processed the data. VD, JTE, and JK analyzed the data. All authors collectively designed the study and contributed to the manuscript text.

## Funding

VD’s involvement in this research was funded by Mitacs Accelerate Postdoctoral fellowship. The research costs associated with this study were funded by a Discovery Grant to JTE from the Natural Sciences and Engineering Council of Canada. JK was funded by Grants-in-Aid for Scientific Research from the Japan Society for the Promotion of Science (20H04568).

## Conflict of Interest

The authors declare that the research was conducted in the absence of any commercial or financial relationships that could be construed as a potential conflict of interest.

## Publisher’s Note

All claims expressed in this article are solely those of the authors and do not necessarily represent those of their affiliated organizations, or those of the publisher, the editors and the reviewers. Any product that may be evaluated in this article, or claim that may be made by its manufacturer, is not guaranteed or endorsed by the publisher.

## Footnotes

^1^ https://www.mturk.com

^2^ https://www.chicagofaces.org/
