# Supplementary material for: The Attractiveness of Masked Faces Is Influenced by Race and Mask Attitudes
Source: Front Psychol. 2022 May 17;13:864936. doi: 10.3389/fpsyg.2022.864936 (PMC9152543; doi:10.3389/fpsyg.2022.864936)
Supplement: Supplementary file 1 [file Data_Sheet_1.pdf]

Supplementary Analyses

Baseline attractiveness ratings

The Asian faces used in this study were the same faces used by Miyazaki & Kawahara (2016). In that study, a total of 1957 images were rated by 30 Japanese raters, in advance of the main study, and then divided into three groups by choosing 22 images with highest attractiveness scores, 22 images with lowest attractiveness scores, and 22 images from the middle of the distribution. Of these 66 images we randomly selected 48 (16 from each category). The descriptive statistics for the ratings made on images used in the present study are shown in Supplementary Table 1. These data show that there was little to no overlap between ratings given to faces in each of these three categories.

The White faces used in this study were taken from Chicago Face Database (Ma, Corell, & Wittenbrink, 2015). The images in Ma et al. (2015) were rated by a large sample of participants, comprised of multiple races, and on multiple scales that included attractiveness. We selected 16 images from this sample with the highest attractiveness scores, 16 images with lowest scores, and 16 images from the middle of the distribution. The descriptive statistics for the ratings made on images used in the present study are shown in Supplementary Table 1. These data show that there was little to no overlap between ratings given to faces in each of these three categories.

Supplementary Table 1: Mean attractiveness ratings (standard deviations) of faces that were used in the present study. Asian faces were selected from the larger sample rated in Miyazaki & Kawahara (2016), as shown in the first row. White faces were selected from the larger sample rated in the Chicago Face Database (Ma et al., 2015) in the second row. The scales used in these previous studies were standardized to a 0-100 point scale (third row). The fourth and fifth rows show the mean attractiveness ratings for un-occluded faces in the present study (Experiment 1: mask; Experiment 2: notebook).

Race	Asian			White		
Attractiveness	Low	Med.	High	Low	Med.	High
Miyazaki & Kawahara, 2016 (0-100 rating scale)	19.78 (12.5)	37.95 (17.52)	59.15 (18.86)			
Chicago Face Database (1-5 rating scale)				2.3 (0.15)	3.12 (0.09)	4.11 (0.31)
CFD converted to 0-100 scale				32.5	53	77.75
Experiment 1, unmasked faces (0-100 rating scale)	46.29 (20.57)	62.22 (15.11)	70.14 (13.07)	36.37 (16.13)	47.7 (12.76)	64.34 (12.3)
Experiment 2, un-occluded faces	45.34 (23.61)	60.28 (12.79)	69.6 (10.3)	40.35 (21.73)	51.83 (16.68)	66.8 (13.5)

Experiment 2

ANOVA analysis of rating scores

The main figure shown in the paper for this experiment (Figure 4) highlights only the difference scores (difference in rated attractiveness for mask versus unmasked faces). Here we report the full mixed model ANOVA in parallel with our treatment of the same data in Experiment 1.

Figure S1 is the parallel figure to Figure 2 in the main text and shows mean attractiveness ratings as a function of the within-participant factors of attractiveness level (low, medium, high), and notebook occlusion (off, on), and the between-participant factor of race of face (White, Asian). A mixed model ANOVA of these ratings showed an expectedly strong effect of attractiveness, $F(2,146) = 84.87$, $p < .001$, $\eta_p^2 = .538$. The main effect of attractiveness was qualified by an interaction with mask, $F(2,146) = 29.04$, $p < .001$, $\eta_p^2 = .285$, reflecting that the addition of an occlusion reduced ratings for highly attractive faces and increased ratings for unattractive faces. The main effect of notebook was not significant, $F < 1$, $p > .7$.

The effect of race was not reliable, $F(1,73) = 1.4$, $p = .24$, nor was the interaction between attractiveness and race, $F(2,146) = 2.7$, $p = .071$. Crucially, race was not involved in any interactions with notebook occlusion, *Fs* < 2, *ps* > .17, reflecting that notebook occlusion did not affect Asian and White faces differently.

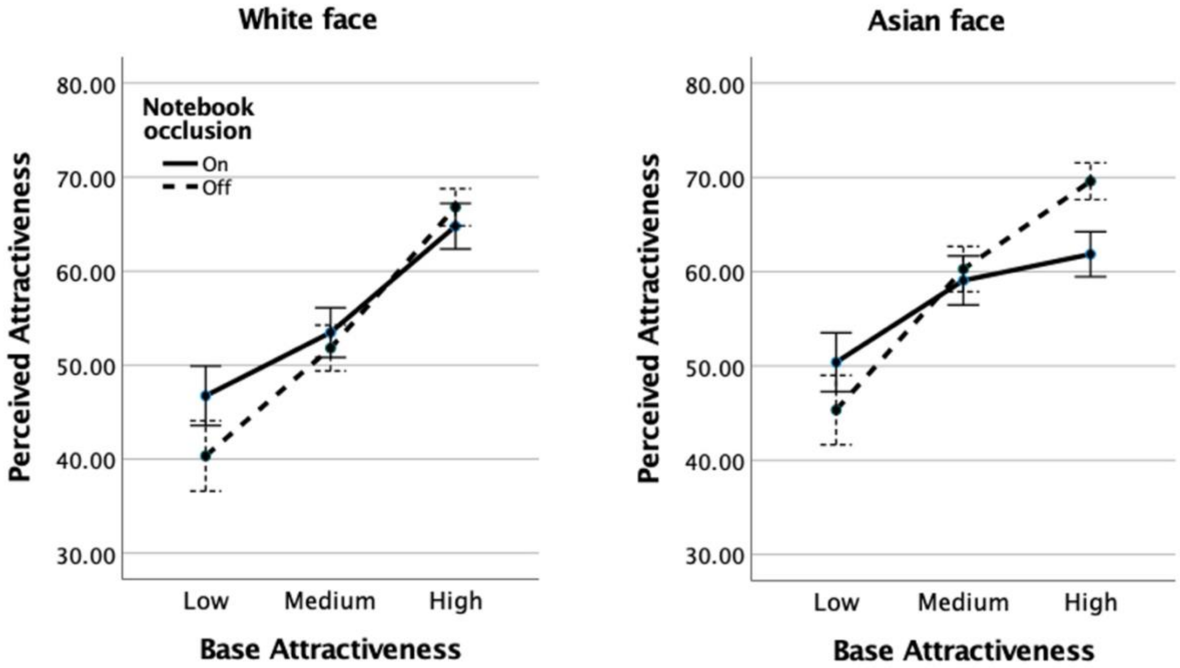

Figure S1: Mean attractiveness ratings for White and Asian faces of low, medium and high base attractiveness, when covered with a notebook (solid line) or not (dashed line). Error bars represent +/- 1 standard error.
